# Prediction of solid-to-solid phase transition for risk assessment of solid forms using quantum mechanical solid-state computations

**DOI:** 10.1063/4.0000920

**Published:** 2025-10-27

**Authors:** Satish Iyemperumal, Charlene Tsay, Monika Warzecha, Ales Medek, Kevin Gagnon, Jiahui Chen

**Affiliations:** Vertex Pharmaceuticals Inc., Boston, MA, USA

## Abstract

Solid form discovery and characterization are critical in pharmaceutical development, as they influence API form selection which can directly impact stability, bioavailability, and manufacturability. In this study, we utilize orthogonal tools to evaluate (i) the risk profile of the stability of a lead crystalline form of an API and (ii) to help prioritize isostructural solvate families that are easier to desolvate to make the desired neat form, since the API is a prolific solvate former. Solid-state computations are leveraged here to improve our understanding of the solid-to-solid phase transition likelihood and impact on their stabilities. For both these assessments, we demonstrate how we integrated computational materials science tools, crystal structures (predicted and experimental), and experimental solid form characterization methods to better understand our material through structure-property relationships.

Firstly, using crystal structure prediction (CSP), we found that there were additional predicted polymorphs similar in stability to our lead form. Density functional theory (DFT)-based predictions showed that the new forms could be made at higher pressures, which was experimentally confirmed. Secondly, since the primary mechanism of making the neat desired form involved desolvation, we wanted to understand if methods such as DFT-based desolvation energies from the crystal structures can help predict general trends of certain solvent systems having an easier/faster desolvation than other solvent systems. We were able to validate the predictions for two different forms.

Overall, given the utility of predictive computational materials science tools, we built workflows to streamline future requests where we can more systematically validate and improve error metrics over a longer period of validation.

